# PFO-spectrum disorder: two different cerebrovascular diseases in patients with PFO as detected by AI brain imaging software

**DOI:** 10.3389/fneur.2024.1357348

**Published:** 2024-02-19

**Authors:** Raluca Ștefania Badea, Sorina Mihăilă-Bâldea, Athena Ribigan, Anca Negrilă, Nicolae Grecu, Andreea Nicoleta Marinescu, Florina Antochi, Cristina Tiu, Dragos Vinereanu, Bogdan Ovidiu Popescu

**Affiliations:** ^1^Department of Neurology, University and Emergency Hospital, Bucharest, Romania; ^2^University of Medicine and Pharmacy Carol Davila, Bucharest, Romania; ^3^Department of Cardiology and Cardiovascular Surgery, University and Emergency Hospital, Bucharest, Romania; ^4^Department of Radiology, University and Emergency Hospital, Bucharest, Romania; ^5^Department of Neurology, Colentina Clinical Hospital, Bucharest, Romania

**Keywords:** patent foramen ovale, stroke, transesophageal echocardiography, contrast transcranial Doppler, magnetic resonance imaging, cerebral white matter lesions

## Abstract

**Background:**

Patent foramen ovale (PFO) is a prevalent cardiac remnant of fetal anatomy that may pose a risk factor for stroke in some patients, while others can present with asymptomatic white matter (WM) lesions. The current study aimed to test the hypothesis that patients with a PFO who have a history of stroke or transient ischemic attack, compared to those without such a history, have a different burden and distribution of cerebral WM hyperintensities. Additionally, we tested the association between PFO morphological characteristics and severity of shunt, and their impact on the occurrence of ischemic cerebral vascular events and on the burden of cerebral WM lesions.

**Patients and methods:**

Retrospective, case–control study that included patients with PFO confirmed by transesophageal echocardiography. Right-to-left shunt size was assessed using transcranial Doppler ultrasound. Cerebral MRIs were analyzed for all participants using the semi-automated Quantib NDTM software for the objective quantification of WM lesions. WM lesions volume was compared between patients with and without a history of stroke. Additionally, the anatomical characteristics of PFOs were assessed to explore their relation to stroke occurrence and WM lesions volume.

**Results:**

Of the initial 264 patients diagnosed with PFO, 67 met the inclusion criteria and were included in the analysis. Of them, 62% had a history of PFO-related stroke/TIA. Overall burden of WM lesions, including stroke volume, was not significantly different (*p* = 0.103). However, after excluding stroke volume, WM lesions volume was significantly higher in patients without stroke (0.27 cm^3^, IQR 0.03–0.60) compared to those with stroke/TIA (0.08 cm^3^, IQR 0.02–0.18), *p* = 0.019. Patients with a history of PFO-related stroke/TIA had a tendency to larger PFO sizes by comparison to those without, in terms of length and height, and exhibited greater right-to-left shunt volumes.

**Discussion:**

We suggest that PFO may be associated with the development of two distinct cerebrovascular conditions (stroke and “silent” WM lesions), each characterized by unique imaging patterns. Further studies are needed to identify better the “at-risk” PFOs and gain deeper insights into their clinical implications.

## 1 Introduction

Patent foramen ovale (PFO) represents a prevalent cardiac remnant of fetal anatomy, being present in about 25% of the general population based on the average of autopsy and transesophageal echocardiography (TEE) studies (1). It is an established risk factor for ischemic stroke, especially cryptogenic stroke in young patients (2). Recently, cerebral WM hyperintensities have been reported in patients with PFO (3, 4). However, the prevalence, underlying pathophysiology, and clinical implications of these lesions in patients with PFO remain an area of ongoing debate.

The current study aimed to test the hypothesis that patients with a PFO who have a history of stroke/TIA, compared to those without such a history, have a different burden and distribution of cerebral WM hyperintensities. Additionally, we tested the association between PFO morphological characteristics and severity of shunt, and their impact on occurrence of ischemic cerebral vascular events and on the burden of cerebral WM lesions.

## 2 Materials and methods

### 2.1 Patient population

**Figure 1:**
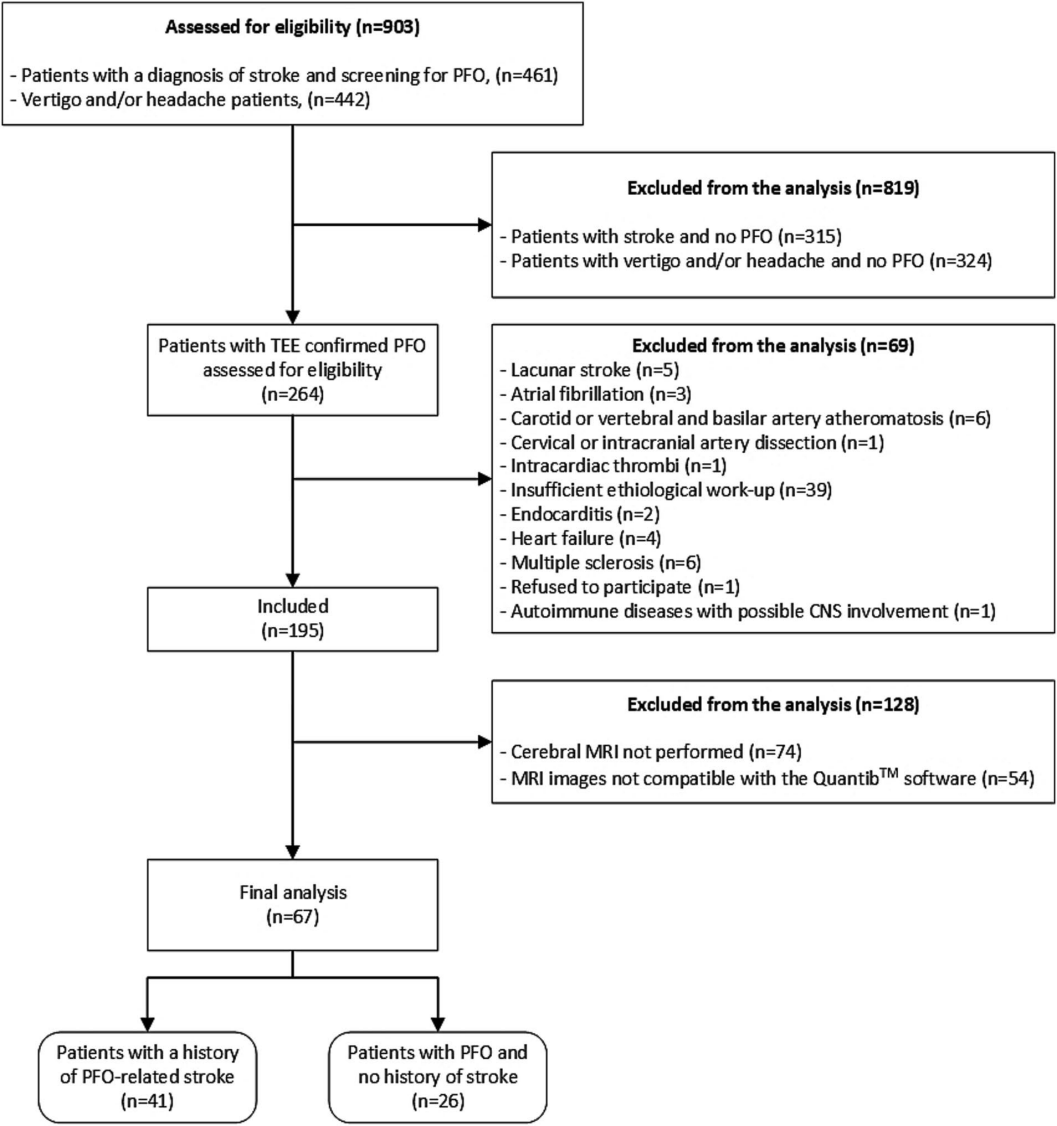
Patient selection flowchart. TEE, Transesophageal echocardiography; PFO, Patent foramen ovale; CNS, Central nervous system; and MRI, Magnetic resonance imaging.

This is a retrospective, case-control, single center study. Data were collected from the electronic medical records of patients admitted between October 2019 and December 2022. The study population included consecutive individuals aged 18–65 years, who presented with either stroke or transient ischemic attack (TIA) associated to a PFO diagnosed by TEE. Patients with other potential stroke or TIA etiologies, as suggested by 24-h ECG monitoring, cervical and cerebral vascular imaging, transthoracic and transesophageal echocardiography, positive work-up suggesting vasculitis, systemic diseases with possible central nervous system (CNS) involvement, including antiphospholipid syndrome, and those with lacunar strokes confirmed by magnetic resonance imaging (MRI) studies were excluded from the study. The control group was selected from consecutive patients referred to or hospitalized during the same time period in our department who underwent brain MRI for vertigo or headache, with no history of stroke/TIA, inflammatory CNS disorders, or other systemic disorders with possible CNS involvement. For vertigo, we included only patients having persistent symptoms for at least 24 h, in order to exclude possible TIAs presenting as vertigo. They were subsequently invited to participate in the study and undergo contrast-enhanced transcranial Doppler (c-TCD) and transesophageal echocardiography (TEE). Patient selection flowchart is presented in Figure 1; a detailed description of the inclusion and exclusion criteria used in the study is presented in the Supplementary material. All patients were required to have undergone cerebral MRI within the preceding month or during their hospitalization, with a quality that met the technical specifications required by the semi-automated software Quantib ND™ (Quantib B.V., Rotterdam, Netherlands), for quantifying WM hyperintensities volume. To avoid omitting lesion visualization in patients with PFO-related stroke/TIA, only MRIs that were performed more than 24 h after symptom onset were included. All patients had MRIs performed within 3 weeks of symptom onset in order to ensure that DWI and ADC sequences show potential subacute or early chronic lesions suggestive of stroke. All patients underwent TEE and contrast transcranial Doppler. Comprehensive morphological TEE characteristics of the PFO, such as lengths and heights, were available for a subset of individuals; severity of right-to-left shunt (RLS) was documented for all participants. Two groups were defined: patients with a history of PFO-related stroke/TIA, and patients with PFO and no history of stroke/TIA. Demographic data, cardiovascular and cerebrovascular risk factors, including hypertension, diabetes, smoking status, chronic kidney disease, and dyslipidemia were collected.

The study was approved by the Local Ethic Committee. All patients signed an informed consent. Research was performed in accordance with the Declaration of Helsinki and GDPR standards.

### 2.2 Transesophageal echocardiography

Transesophageal echocardiography was conducted using an E95 echo machine from General Electric (GE) Healthcare, Horten, Norway, equipped with a dedicated TEE probe. A single examiner (SMB) conducted all examinations. Interatrial septum was analyzed using dedicated views, including long-axis, short-axis, and bicaval views. These views were used to assess the presence of a PFO, and to identify any color shunt at this level. Length of the PFO was measured as the longest distance of the overlap between the ostium primum and ostium secundum, while the height of the PFO was defined as the maximum distance between these two structures. Measurements of PFOs lengths and heights were available in a limited number of patients due to the retrospective nature of data collection. All patients underwent a “bubble test” for the assessment of RLS, using intravenous administration of agitated saline solution, at rest and after intense Valsalva maneuver; RLS was assessed by the presence of microbubbles passing through the PFO. Additionally, presence of an interatrial septal aneurysm was assessed, defined as an excursion of more than 10 mm of the interatrial septum. Other anatomical features, such as a prominent Eustachian valve or a prominent Chiari’s network, were also assessed.

### 2.3 Contrast transcranial Doppler

Contrast transcranial Doppler was performed using a Digital DWL® TCD device equipped with DWL® routine software (Doppler-Box™, Compumedics Germany GmbH, Singen, Germany). A single examiner (RȘB) conducted all examinations. Standard protocol involved insonating both middle cerebral arteries (MCAs) at a depth of 55 mm, using 2 MHz pulsed wave (PW) monitoring probes affixed to a DiaMon® headset (Compumedics Germany GmbH, Singen, Germany). A 23-gauge cannula was inserted into one of the antecubital veins, and a blood sample of 1 mL was withdrawn from the patient. The blood sample was then mixed with 1 mL of air and 8 mL of 0.9% saline solution. The resulting mixture was agitated 10 times using two syringes and a three-way stopcock before being injected into the intravenous cannula. Subsequently, the patient was instructed to perform two Valsalva maneuvers. The total count of microbubbles passing through both MCAs during the entire test (during resting and during the two Valsalva maneuvers) was recorded, and the severity of the RLS was assessed using Spencer logarithmic scale (SLS), as follows: grade 0, no microembolic signals (MES); grade 1, 1–10 MES; grade 2, 11–30 MES; grade 3, 31–100 MES; grade 4, 101–300 MES; and grade 5, over 300 MES (curtain pattern) (5).

### 2.4 Magnetic resonance imaging

**Figure 2:**
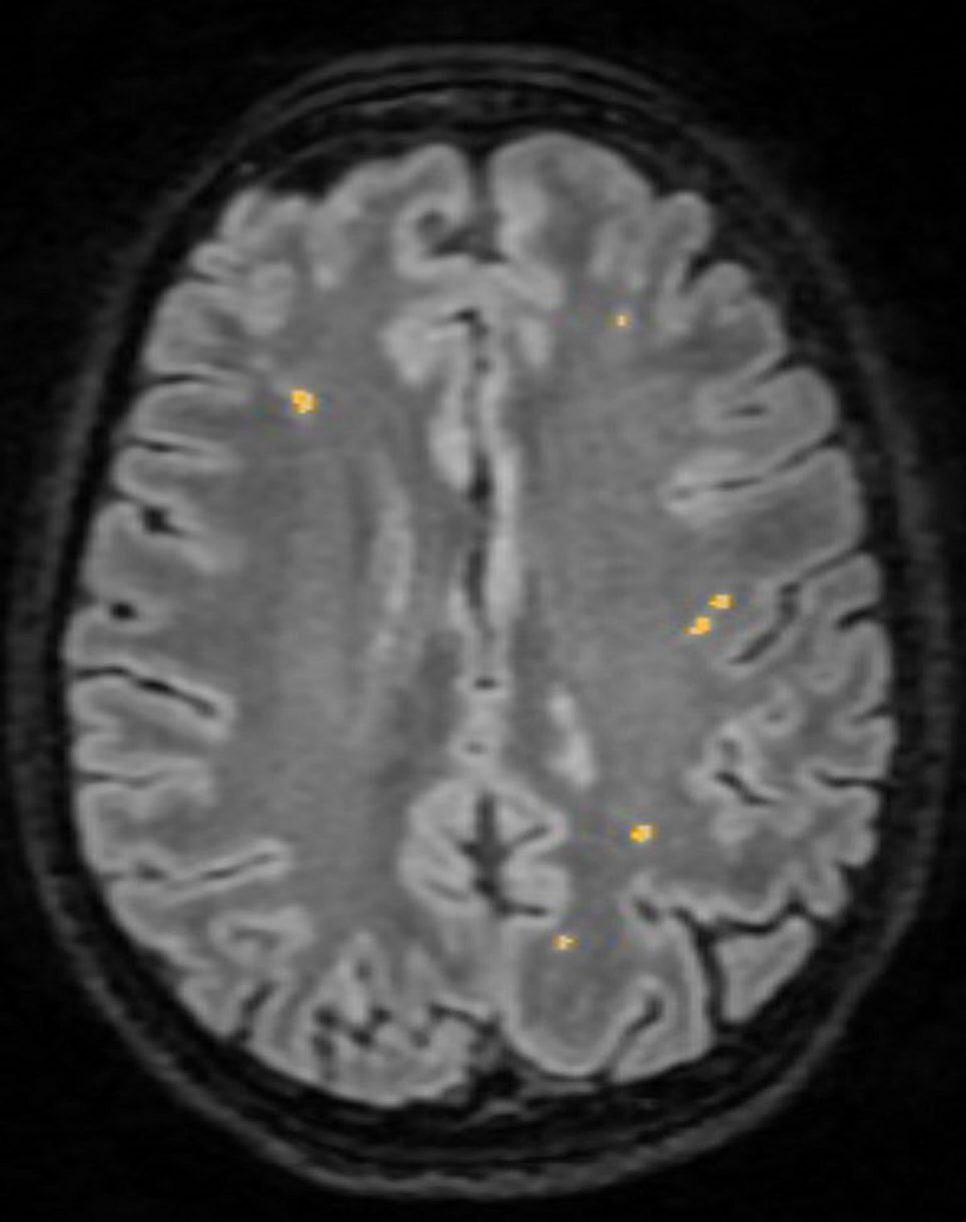
White matter lesion identification using Quantib ND™.

In order to be compatible with the image processing software and thus accepted in the study, MRI studies had to be performed on either 1.5 or 3 Tesla MRI machines, with a non-contrast enhanced 3D T2-FLAIR acquisition with an inversion time of 2,250 ms, an echo time of 140 ms, a voxel size ranging between 1 and 3, and a slice thickness of 1.5–3 mm; patients with examinations utilizing filters such as GE filter SCIC and GE filter E were excluded from the study due to incompatibility with the software. MRI images were assessed using the semi-automated software Quantib ND™ to measure WM lesional volume. Quantib ND™ was selected for its advanced artificial intelligence (AI) algorithms, which facilitate accurate and efficient quantification of brain lesions. The process involved uploading the MRI scans into the software, where Quantib ND’s AI algorithms semi-automatically identified and measured the WM lesions. Any necessary manual adjustments were made to ensure accuracy, following which the software provided quantitative data on lesional volume. This data were then extracted for statistical analysis. For each of the 3D FLAIR acquisitions, each of the T2 weighted hyperintensity, surrounded by normal isointense WM, was marked (Figure 2). Lesions located in the basal ganglia and brainstem were excluded from measurement, as they were ascribed to small vessel disease. Measurement protocol for patients with stroke comprised two phases: initially, all cerebral lesions were automatically identified and measured, including the volume of the stroke; subsequently, the volume of the stroke was excluded, and the remaining volumes were recorded. Two examiners, a trained neurologist (RȘB) and a radiologist (ANM), conducted all examinations.

### 2.5 Statistical analysis

Normality of data distribution for continuous variables was assessed using the Shapiro–Wilk test. Continuous variables with a normal distribution were presented as mean ± standard deviation, while those with a non-normal distribution were reported as median (interquartile range). Categorical variables were presented as rates (percentages). To compare differences between groups, independent *t*-test was used for continuous variables, while χ^2^ or Fisher’s exact test was used for categorical variables. To determine differences in WM lesions volume between groups, the Mann–Whitney U test was conducted. Additionally, a Quade nonparametric ANCOVA test was employed to assess differences in WM lesions volumes between groups, while controlling for the following variables: age, gender, hypertension, glomerular filtration rate, LDL-cholesterol, and triglycerides blood levels. To assess the relationship between morphological characteristics (height and length) of the PFO and WM lesional volume, Spearman’s rank-order correlation test was conducted. Missing values were left in for the analysis. This decision was based on the nature of the missing data, which was determined to be missing at random (MAR), and our assessment that their inclusion would not significantly bias the results. Inter-observer reliability between the two estimations of WM lesion volume, using the semi-automated software Quantib™, was determined using the Intraclass Correlation Coefficient (ICC, two-way mixed, absolute agreement). A *p* value of less than 0.05 was considered statistically significant. Statistical analysis was performed using IBM SPSS Statistics for Windows, Version 29.0. (Armonk, NY: IBM Corp).

In order to understand the sensitivity of our study, a *post hoc* power analysis was conducted, considering the observed effect size, the sample sizes of the two groups, and the alpha level. The effect size (Cohen’s *d*) was derived from the mean difference in the total volume of WM lesions (without the stroke volume in cases of patients with a history of stroke) between patients with and without a history of stroke/TIA, divided by the pooled standard deviation of both groups. The post-hoc power analysis revealed that the study achieved a power of 88.31%. This suggests that, given the observed effect size, our study had an 88.31% chance of detecting a true effect of this magnitude, assuming it exists. Additionally, in a retrospective evaluation to address the adequacy of our sample size, we estimated the required number of participants per group to achieve a power level of 80% with an alpha level of 0.05, based on the observed effect size (Cohen’s *d*) of 0.802 from our study. This estimation suggested that approximately 26 participants per group would be necessary to adequately power a study to detect the differences in the total volume of WM lesions between patients with and without a history of stroke/TIA. Statistical power calculations were performed using G*Power software (Version 3.1.9.7, Heinrich Heine University Düsseldorf, Germany). (6)

## 3 Results

### 3.1 Patient population

After reviewing the medical records from October 2019 to December 2022, a total of 461 patients aged 18–65 years, diagnosed with stroke and screened for PFO were initially identified in the neurology department. Among these, 146 patients were confirmed to have PFO. After applying the exclusion criteria, 45 patients were found to have other potential causes of stroke or had insufficient etiological work-up and were excluded from the study. An additional 60 patients were excluded because they either did not undergo cerebral MRI or had MRIs that were incompatible with the Quantib™ software specifications. Consequently, the final sample comprised 41 patients diagnosed with PFO-attributable stroke.

For the control group, 442 patients who were evaluated in the neurology department for headache and/or vertigo were identified from medical records and invited for PFO screening. Of these, 118 patients received a confirmed PFO diagnosis. Subsequently, 23 patients were excluded due to diagnoses of other conditions that could result in cerebral lesions, and one patient declined participation. Furthermore, 68 patients were excluded for not undergoing a cerebral MRI or having an MRI incompatible with the Quantib™ software. Ultimately, 26 patients, with a PFO diagnosis but no history of stroke, were included in the control group (Figure 1).

Demographic characteristics of the groups are reported in Table 1. There were no statistically significant differences, apart from gender.

**Table 1:** Comparison of demographic, PFO, and cerebral MRI characteristics between groups.

Characteristics	Patients with PFO-related stroke/TIA		Patients with PFO and no history of stroke/TIA		*p*
*n*/total (%)	41/67 (61.19)		26/67 (38.81)		
Age	39.95 ± 11.43		40.07 ± 9.39		0.961
Gender (*n*)	Female	Male	Female	Male	0.011^*^
	18	23	20	6	
Hypertension (*n*, %)	10/41 (24.40)		3/26 (11.50)		0.225
Diabetes (*n*, %)	0		1/41 (2.40)		1
LDL cholesterol (mg/dL)	110.22 ± 44.17		115.79 ± 34.77		0.606
Triglycerides (mg/dL)	83.00 (60.25–124.50)		85.00 (28.00–127.50)		0.467
Glomerular filtration rate (by CKD-EPI) mL/min/1.72 m^2^	113.49 (96.10–121.67)		114.48 (102.46–115.01)		0.457
Smoker (*n*, %)	13/41 (31.70)		12 (46.20)		0.302
PFO characteristics					
PFO length (mm)^†^	7.00 (3.00–11.00)		7.00 (2.25–8.75)		0.294
PFO height (mm)^†^	3.00 (2.00–4.00)		2.00 (2.00–3.50)		0.407
Interseptal aneurysm (*n*, %)	7/41 (17.10)		4/26 (15.40)		1
Chiari’s network (*n*, %)	0		1/26 (3.80)		0.388
Prominent Eustachian valve (*n*, %)	1/41 (2.40)		2/26 (7.70)		0.555
RLS shunt severity (SLS estimated on c-TCD)	3.00 (2.00–5.00)		2.00 (1.00–4.00)		0.134
Cerebral MRI characteristics					
Stroke volume cm^3^	1.85 (0.90–7.82)		NA		NA
Total WM lesional volume (including stroke) cm^3^	0.43 (0.10–2.20)		0.27 (0.03–0.60)		0.103
WM lesional volume (excluding stroke volume) cm^3^	0.08 (0.02–0.18)		0.27 (0.03–0.60)		0.019*

^*^Statistical significance was considered at a *p* value less than 0.05. ^†^Measurements of PFO length and height were available in only 47 patients: 31 patients with PFO-associated stroke, and 16 patients with PFO and no history of stroke. c-TCD, Contrast transcranial Doppler; CKD-EPI, Chronic Kidney Disease Epidemiology Collaboration; PFO, Patent foramen ovale; RLS, Right-to-left; SLS, Spencer logarithmic scale; MRI, Magnetic resonance imaging; and WM, White matter.

### 3.2 White matter lesional volume

**Figure 3:**
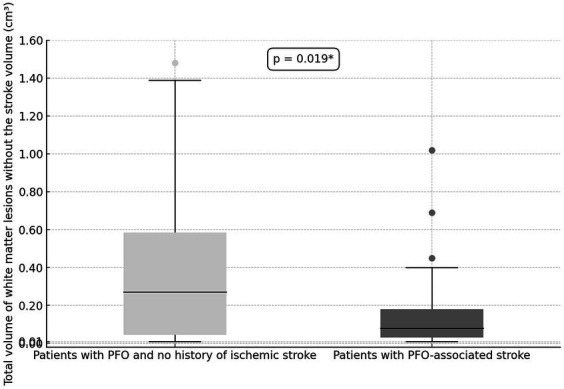
Comparison of total WM lesions volume (excluding the stroke volume) between patients with PFO without a history of stroke/TIA and those with a history of PFO-related stroke/TIA.

When comparing the overall burden of WM lesions, which includes the stroke volume, the lesional volumes appear similar between the two groups. However, upon excluding the stroke volume from the analysis, patients with PFO with no history of stroke displayed significantly higher volumes of WM lesions (0.27 cm^3^, IQR 0.03–0.60) compared to patients with PFO-related stroke/TIA (0.08 cm^3^, IQR 0.02–0.18), *p* = 0.019 (Figure 3). This statistically significant difference persisted even after controlling for cardiovascular risk factors (*F* = 7.27, *p* = 0.010). The inter-observer agreement in the assessment of WM lesions volume in patients with PFO was excellent among the two observers (ICC = 0.980).

### 3.3 PFO characteristics and cerebral lesions

**Figure 4:**
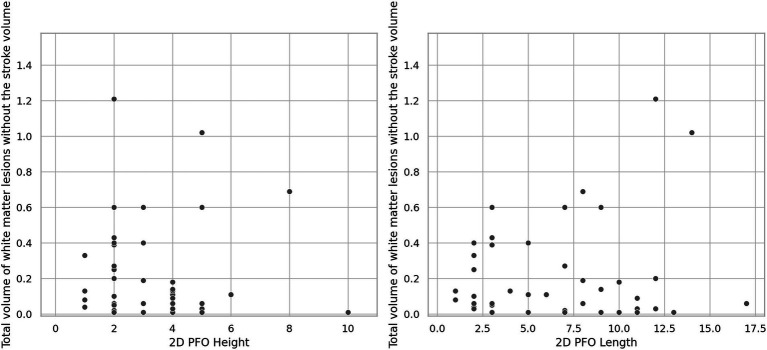
Relationship between PFO height and length and cerebral WM lesions volumes (excluding stroke volumes).

Patent foramen ovales morphological features are presented in Table 1. There was a tendency of patients with PFO-related stroke / TIA to have larger PFOs and greater shunts than patients with no history of stroke/TIA (Table 1). Meanwhile, presence of interatrial septal aneurysms, Chiari’s network, or of a prominent Eustachian valve were not different between groups (Table 1), and they did not contribute to the burden of cerebral WM lesional volume (Table 2). There was no significant correlation of cerebral WM lesions volume (excluding stroke volume) and the PFOs’ lengths [*ρ*(45) = −0.139, *p* = 0.351], heights [*ρ*(45) = 0.005, *p* = 0.972] or shunt size [*ρ*(65) = −0.115, *p* = 0.372] (Figure 4).

**Table 2:** WM lesion volume by anatomical features of the PFO.

	WM lesional volume (without the volume of stroke) cm^3^		*p*	*U*
	Present	Absent		
Interseptal aneurysm	0.18, 0.01–0.54	0.22, 0.04–0.27	0.99	307
Chiari’s network^*^	1.39	0.11, 0.03–0.28	0.10	-
Prominent Eustachian valve	0.27, 0.17–0.74	0.11, 0.03–0.29	0.31	130

^*^For WM lesion volume based on the presence of Chiari’s network, there was only one data point for the “Present” group.

## 4 Discussion

Our study confirmed the hypothesis that patients with PFO and no history of stroke/TIA have significantly more severe WM lesions than patients with a history of stroke/TIA related to PFO, after excluding from the analysis the stroke volume itself. These findings suggest that the presence of a PFO may be a risk factor for the development of two distinct cerebrovascular diseases: stroke and “silent” WM lesions, each characterized by unique imaging patterns and conceivably distinct pathophysiological mechanisms.

Previous reports showed that patients with PFO may have silent cerebrovascular ischemic lesions. While some authors strictly acknowledge the presence of WM lesions in patients with PFO (7–9), others have described potential patterns of these lesions, such as asymmetric distribution in the subcortical and periventricular regions (10), or a predominant involvement of the frontal (11) or occipital lobes (12). Such WM lesions were described in various populations with PFO, but studies focused mainly on patients with various comorbidities, such as pulmonary embolism (13) or migraine (12). On the other hand, a large community-based cohort study conducted by Di Tullio et al. (14) failed to establish an association between PFO and stroke or subclinical cerebrovascular disease.

In our study, considering the relatively limited volume of background WM lesions observed in patients with PFO-associated stroke/TIA when compared to those without a stroke history, one might postulate that patients with a high burden of silent WM lesions are less likely to experience clinically significant PFO-related strokes, implying the existence of divergent underlying pathophysiological mechanisms for these two conditions.

The most accepted hypothesis of potential stroke mechanisms in patients with PFO are paradoxical embolism allowing venous thrombi to pass into the cerebral arterial circulation (15), cardiac arrythmias induced by abnormal cardiac conduction pathways (16), or *in situ* clot formation (17) favorized by high risk anatomical features of the PFO. Nevertheless, recent studies have described additional factors that may contribute to the occurrence of PFO-associated cerebrovascular disease. Notably, patients with PFO have been found to exhibit endothelial dysfunction as evidenced by alterations in the L-arginine/ADMA ratio (18), reduced fibrinolytic activity (19), but also increased levels of total circulating homocysteine serum levels (20).

The pathophysiology underlying “silent” white matter lesions in patients with RLS presents a compelling area of study, particularly concerning the role of cerebral hemodynamics. It has been proposed that patients with RLS may exhibit varying degrees of cerebrovascular reactivity (CVR), which could influence the burden of WM lesions. While some studies suggest that CVR has no role in the WM lesional burden in patients with RLS (21), other research indicates that patients with RLS who exhibit WM lesions tend to have lower CVR compared to those without RLS or WM lesions (22). Additionally, a decreased CVR was also shown to be linked to WM lesional development in patients with migraine, but in these patients, age was the major determinant of the WM lesional volume (23).

Whether there is a “protective” predisposition that prevents large, symptomatic stroke, but allows for the accumulation of subclinical microinfarcts, or whether there is an unknown precipitating factor that leads to stroke in an otherwise apparently previously asymptomatic PFO are hypothesis for future studies and would delineate these potential distinct facets of a potential PFO-associated cerebral disease spectrum. “Silent” WM lesions are clinically relevant, since it has been demonstrated that these are associated with varying degrees of cognitive impairment, ranging from mild cognitive impairment (24) to dementia (25). However, to better understand the long-term cognitive consequences of extensive WM disease in patients with PFO, more prospective studies are required.

Previous reports have indicated that there is probably a relationship between certain morphological characteristics of PFO and the risk of stroke recurrence, albeit with somewhat conflicting results. They have assessed, depending on echocardiography protocol, various parameters, and most agree that greater PFO height and length (26), the presence of an atrial septal aneurysm (27), or a more severe right-to-left shunt (28) are more commonly found in patients with PFO and cerebrovascular events. Our findings are in agreement with regard to PFO height and length, as well as shunt size, but did not reach statistical significance; this could be due to the small sample size, since there were no differences between the overall demographic characteristics of our cohort and those from other reports. An alternative explanation, as suggested by other researchers (29, 30), is that these anatomical characteristics are purely innocent bystanders, and PFO-related stroke might occur through more complex, yet still not understood, mechanisms.

There are several limitations to our study. Firstly, the retrospective design and recruitment of participants from a single center might limit the generalizability of our findings. Meanwhile, the retrospective design made standardization of examinations impossible. Consequently, a considerable number of patients had to be excluded from the analysis due to the inadequate quality of their MRI images, making them incompatible with the Quantib™ system’s analytical capabilities; furthermore, the difference in magnetic field strength and slice size can also impact lesion number and size analysis. Secondly, the control group was not specifically matched for age and gender, since the control group was selected from consecutive subjects meeting the specified inclusion and exclusion criteria. Additionally, while a *post hoc* power analysis was performed, it is important to acknowledge that such retrospective evaluations inherently reflect the characteristics of the observed data, rather than pre-study expectations. Despite these limitations, our findings offer preliminary insights into the differences in WM lesions volumes between patients with and without a history of PFO-associated stroke/TIA. Our methodology was robust, with the use of high-quality MRI scans and rigorous criteria for patient selection. These results serve as an impetus for larger studies.

In conclusion, we compared the cerebral WM lesions volume in patients with PFO with and without a previous stroke/TIA, using semi-automated AI-derived volumetry software. The major finding of our study is that PFO is a risk factor not only for clinically manifest ischemic stroke, but also for extensive cerebral WM lesions. Additionally, these results highlight the value of AI-derived volumetry software in finding new biomarkers of cerebral disease in patients with PFO.

## Data availability statement

The raw data supporting the conclusions of this article will be made available by the authors, without undue reservation.

## Ethics statement

The studies involving humans were approved by the Local Ethics Committee of the University and Emergency Hospital of Bucharest. The studies were conducted in accordance with the local legislation and institutional requirements. The participants provided their written informed consent to participate in this study.

## Author contributions

RB: Conceptualization, Data curation, Investigation, Methodology, Visualization, Writing – original draft, Writing – review & editing. SM-B: Conceptualization, Data curation, Formal Analysis, Investigation, Writing – review & editing. AR: Conceptualization, Investigation, Methodology, Project administration, Writing – review & editing. AN: Visualization, Writing – review & editing. NG: Writing – original draft, Writing – review & editing. AM: Data curation, Formal Analysis, Investigation, Writing – review & editing. FA: Conceptualization, Writing – review & editing. CT: Methodology, Project administration, Writing – review & editing. DV: Data curation, Investigation, Writing – review & editing. BP: Conceptualization, Formal Analysis, Methodology, Project administration, Supervision, Writing – review & editing.
