# Supplementary material for: PFO-spectrum disorder: two different cerebrovascular diseases in patients with PFO as detected by AI brain imaging software
Source: Front Neurol. 2024 Feb 19;15:1357348. doi: 10.3389/fneur.2024.1357348 (PMC10909929; doi:10.3389/fneur.2024.1357348)
Supplement: Supplementary file 1 [file Table_1.DOCX]

Supplementary material – Inclusion and Exclusion criteria

| **Study group:** |
| --- |
| **Inclusion criteria:** |
| 1. Age between 18 and 65 years |
| 1. Diagnosis of PFO associated stroke or TIA |
| 1. Cerebral MRI study compatible with the Quantib ND^TM^ software performed within 3 weeks of the neurological symptoms’ onset |
| **Exclusion criteria:** |
| 1. Age <18 years or >65 years |
| 1. Diagnosis of lacunar stroke (defined as stroke with a diameter less than 15 mm on cerebral MRI) |
| 1. Any other concomitant condition that may be associated with a risk of stroke: |
| - 1. Cardiac arrythmias (atrial fibrillation or flutter) detected on long-term cardiac rhythm monitoring (at least 24 hours) |
| - 1. Other cardiac malformations (e.g., ventricular septal defect, Ebstein anomaly) |
| - 1. Other arterio-venous shunts (e.g., pulmonary shunts) |
| - 1. Heart failure with reduced ejection fraction (<50%) |
| - 1. Intracardiac tumors |
| - 1. Intracardiac thrombi |
| - 1. Endocarditis |
| - 1. Cervical and cerebral arteries atheromatosis |
| - 1. Arterial dissections |
| - 1. Autoimmune diseases (lupus, antiphospholipid syndrome, Sjögren’s syndrome) |
| - 1. Cerebral or systemic vasculitis |
| - 1. Consumers of illicit drugs |
| 1. Insufficient work-up to establish a stroke etiology (sufficient work-up was defined as: ECG monitoring for a minimum of 24 hours, cervical and cerebral vascular imaging by ultrasound or CT angiography, transthoracic and transesophageal echocardiography) |
| 1. Intracerebral hemorrhage |
| 1. Intracranial or systemic neoplasm |
| 1. Metabolic encephalopathies |
| 1. Active pregnancy |
| 1. Absence of cerebral MRI study performed within the first 3 weeks from the neurological symptoms’ onset |
| **Control group:** |
| **Inclusion criteria:** |
| 1. Age between 18 and 65 years |
| 1. Patients consulted in the Neurology department for headache or vertigo lasting more than 24 h, diagnosed with PFO and without a history of stroke/TIA |
| 1. Cerebral MRI compatible with the Quantib ND^TM^ software, performed within one month prior to their evaluation or during hospitalization |
| **Exclusion criteria:** |
| 1. History of stroke/TIA |
| 1. Evidence of acute stroke on cerebral MRI |
| 1. Patients who were diagnosed with conditions that can lead to cerebral white matter lesions: |
| - 1. Inflammatory/demyelinating diseases of the CNS (multiple sclerosis, anti-MOG disease, neuromyelitis optica spectrum disorder) |
| - 1. Cardiac arrythmias (atrial fibrillation or flutter) detected on long-term cardiac monitoring (at least 24 hours) |
| - 1. Other cardiac malformations (e.g., ventricular septal defect, Ebstein anomaly) |
| - 1. Other arterio-venous shunts (e.g., pulmonary shunts) |
| - 1. Heart failure with reduced ejection fraction (<50%) |
| - 1. Intracardiac thrombi |
| - 1. Intracardiac tumors |
| - 1. Endocarditis |
| - 1. Cervical and cerebral arteries atheromatosis |
| - 1. Arterial dissections |
| - 1. Autoimmune diseases (lupus, antiphospholipid syndrome, Sjögren’s syndrome) |
| - 1. Cerebral or systemic vasculitis |
| - 1. Diagnosis of meningitis/encephalitis (acute or history of meningitis/encephalitis) |
| - 1. Posterior reversible encephalopathy syndrome |
| 1. Active pregnancy |
| 1. Consumers of illicit drugs |
| 1. Intracerebral hemorrhage |
| 1. Intracranial or systemic neoplasm |
| 1. Metabolic encephalopathies |
| 1. Absence of cerebral MRI study performed within the first 3 weeks from the neurological symptoms’ onset |
| 1. Refusal to participate |
| Abbreviations: CNS: central nervous system; MRI: magnetic resonance imaging; PFO: patent foramen ovale; TIA: transient ischemic attack. |
